# Lectures on Diseases of the Skin

**Published:** 1886-11

**Authors:** Henry Wile

**Affiliations:** Lecturer on Dermatology, Atlanta, Ga.


### LECTURES ON DISEASES OF THE SKIN.

DELIVERED AT THE ATLANTA MEDICAL COLLEGE DURING THE SESSION OF 1885–6 BY HENRY WILE, M. D., LECTURER ON DERMATOLOGY, ATLANTA, GA.

### Lecture IX.

SEBORRHŒA CONCLUDED—COMEDO—MILIUM—SEBACEOUS CYSTS—CLASS II. HYPERÆMIAS—ACTIVE—PASSIVE.

Reported Stenographically for The Atlanta Medical and Surgical Journal by W. Kay Tewksbury.

The prognosis is generally favorable where the treatment is perseveringly carried out. When the hair has fallen out in consequence of the long continuance of the disease on the scalp, there is little hope of its being restored. Judicious treatment may, however, save the hair that remains.

In the treatment of seborrhœa, local medication is the more efficient. The internal use of medicines, such as tonics of iron, quinine, cod-liver oil is indicated in cases of general debility, anæmia and scrofulous habit. Arsenic in the form of Fowler's solution, given in doses of three to five minims, diluted with water after meals, is often of service.

It remains for local treatment to dislodge the secondary products of the disease and bring about such changes in the nutrition of the cutaneous tissues as are calculated to restore tone to the sebaceous glands, and return them to the proper discharge of their functions. The first step in the treatment, under every circumstance, is to remove thoroughly all deposits of scales and crusts. When this deposit is scanty a shampoo with castile soap and hot water may suffice. Instead of castile soap the solution already mentioned, as *spiritus saponatus kalinus*, may be used, it having both saponaceous and stimulating properties, besides being an elegant, refreshing preparation. The formula is as follows:

℞. Saponis Viridis........................℥vi
Alcoholis...........................f ℥iii
M. Filtra et adde.
Spirit. lavandulæ......................f ʒii
Sig. Spiritus saponatus kalinus.

When there is considerable accumulation of scales and sebaceous matter, as upon the scalp, it will be expedient to use some oil, as olive oil, anointing the part freely, and allowing it to remain, covered with a flannel cloth for some hours or over-night, then washing the surface with hot water and the above liquid soap. It may be necessary to repeat the procedure, according to the amount and density of the deposit, but by this means the surface can be thoroughly cleansed so that other remedies can be applied with effect.

For the most part, stimulating application will give the best results, and on the scalp, when the hair is harsh and dry and the surface slightly hyperæmic, a combination with some bland oil is indicated, thus: castor oil one-half ounce, balsam Peru ten drops, alcohol four ounces; or salicylic acid one drachm, glycerine two drachms, alcohol four ounces; instead of salicylic acid, boracic acid, chloral or resorcin may be used, the latter being specially indicated when the hair falls out. These lotions are to be applied once daily, and if the surface continues dry some pomade may be rubbed in occasionally. Stimulating ointments are of service, such as ammoniated mercury, twenty to eighty grains to the ounce of vaseline; or oil of cade or tincture rusci, one-half to one drachm to the ounce of vaseline. These lotions and ointments may be rendered more acceptable by the addition of a few drops of oil of rose, rose-geranium, lavander, bergamot, etc. The preparations are to be used diligently for weeks, even months if necessary, in order to establish a healthy action of the sebaceous glands. After a time, if the scaling is markedly diminished, the applications may be made less frequently. When the disease exists in the oily form, as upon the face, about the nose, lotions containing agents that dissolve oil and saponify fat, are indicated; such contain alcohol, ether, glycerine and carbonate of potash.

In seborrhœa of the genitals, absorbent powders, such as oxide of zinc, talc, subnitrate of bismuth, may be employed, together with pledgets of absorbent cotton renewed frequently where surfaces are in apposition.

COMEDO consists of a plug of sebaceous material occupying the duct of the sebaceous gland. The outlet of the gland is elevated or depressed, and the free end of the plug appears capped with a black speck. By pressure with the fingers on each side of the gland, the comedo is brought to the surface, a mould of the duct and varying in size according to the calibre of the duct. In the larger ones it appears as a yellowish or waxy, oval body; in the smaller ones it is white, thin and worm-like. The common seat of the lesion is about the nose, forehead, chin, chest and back. Most every person is more or less affected, but appearing and disappearing, the disease is not noticeable. It is only when the lesions appear and continue in large numbers that they prove disfiguring enough to excite attention. They develop for the most part at the time of puberty, and are as a rule associated with seborrhœa and acne.

The cause of comedo may be sought for in the fact that the horny cells absorb the oily constituents of the sebaceous secretion and this renders the product too solid to escape of itself. Another cause may also exist in a more or less relaxed condition of the muscular structure (arrectores pilorum), by means of which the contents of the glands are expelled. Then again, the cause may be foreign matter that blocks up the outlet, as in the case of workers in tar and paraffine factories. The disorder is frequently associated with gastric trouble and constipation, and in the female with menstrual difficulties.

The comedo, when removed by pressure and examined microscopically, is found to consist of horny epidermal cells enclosing particles of fatty matter, disintegrated cells, amorphous material, and occasionally a lanugo hair. The dark spot at one end is due to particles of dust from the atmosphere. The prognosis is favorable.

The treatment consists first in removing the sebaceous plugs by pressure with the fingers, protected by a thin towel, or by means of a watch-key or instrument (comedo extractor) invented for this purpose. After removal the parts are to be treated with stimulating and fat-extracting preparations. Lotions, containing alcohol, ether, sulphur, glycerine and spirits of soap, are valuable. Frequent bathing in hot water is also advisable. The comedones may re-appear, but they should be expressed, and the treatment continued until they cease to appear.

MILIUM, an affection also due to retention of secretion, appears in the form of small pin-head to hemp-seed sized, white, usually slightly elevated, rounded lesions, found mostly where the skin is thin, as on the eyelids, forehead, temporal regions, corners of the mouth, glans penis and inner surface of the labia minora; also in cicatrices due to burns, lupus or syphilis. It occurs more in females. The seat of the lesion is in one or more acini of the sebaceous glands or in the hair follicle. The outlet of the gland becoming obstructed, a retention of the sebum is caused, which, increasing, gives rise to a distention. Sometimes milium is formed when there is no obstruction, and this is probably due to some perverted action of the gland. Kaposi thinks that in this latter case the cells, instead of undergoing a fatty degeneration, simply dry out, and becoming horny like other epidermal cells are unable to reach the surface. The lesion is separated from the surface of the skin by a thin layer of epidermis, which if incised will give exit to a small, white or yellowish fatty mass. It is composed of epithelial cells, which are sometimes arranged in alveoli surrounded by bands of connective tissue, or the cells become flattened out into layers and on section appear as concentrically arranged lamellæ. The mass may contain cholesterin crystals and often a lanugo hair coiled about.

The treatment ordinarily consists in incising each lesion with a sharp-pointed bistoury and squeezing out its contents.

SEBACEOUS CYSTS (atheroma, wen) are oval or rounded tumors varying in size from a pea to a walnut and larger; found mostly on the forehead and scalp. They are single or multiple and may extend into the subcutaneous connective tissue, even forming connections with the periosteum of the cranium. The lesions occur, as a rule, late in life, are benign, but may be the starting point for cancerous formations. The cyst is surrounded by a capsule of connective tissue, which in recent formations is lined with cylindrical epithelial cells with a subsequent layer of squamous nucleated cells. The great bulk of the cyst is soft, syrupy, white or gray; sometimes it is hard and consists of desquamated cells, compound granule cells, cholesterin plates, which give it a glistening appearance, leucin and tyrosin. Calcification may also take place. According to Rindfleisch, the capsule consists of two or three layers of squamous epithelial cells, and the larger the cyst the thinner the capsule

The treatment consists in a thorough extirpation of the cyst. It should be removed together with its capsule. When the capsule is adherent, the application of iodine to the walls of the cavity is all that is necessary.

HYPERÆMIÆ of the skin, as a class, are the result of certain disturbances of circulation. The pathology of the process has already received attention. It affects, specially, the most superficial cutaneous vessels and is made manifest by a discoloration of the part. The appearance on the surface of the skin is characterized by the presence of, as a rule, non-elevated, circumscribed or diffuse lesions, varying in size from a pin head (maculæ, roseolæ) to large areas (erythema), and in color from pink to dark red or bluish red. A special characteristic of the lesions is their disappearance under pressure with the finger. They may be acute, even evanescent, or chronic, lasting for months, and may be attended with itching or burning. In disappearing they generally leave no trace, but when the process has been unusually active, a slight desquamation of the epidermis may follow, or where it has been persistent, it may leave pigmented spots or lead to inflammatory changes resulting in the development of connective tissue and consequent thickening of the skin.

Hyperæmiæ may be active or passive. In the former the blood-current is more rapid than normal and the local temperature is elevated, while in the latter the current is slower than normal and the temperature is lowered. They may be distinguished by the color, the former being of a bright red, the latter of a dark red or violaceous hue. The active hyperæmiæ are further divided into idiopathic and symptomatic. Idiopathic, active hyperæmiæ are strictly local affections, and are the direct results of local irritation. There are four varieties:

1. *Erythema traumaticum* is due to mechanical irritation, especially friction, as with the finger-nails or brush, or by pressure and friction from wearing apparel or weight of the body of a patient confined to bed for a long time.

2. *Erythema caloricum* is due to exposure to extremes of atmospheric temperature, as the heat of the sun (sun-burn) or cold air; also to hot and cold baths.

3. *Erythema veneuatum* is due to irritations from chemicals applied to the skin, as acids, alkalies, or from poisonous plants, dye-stuffs and medicinal applications, as arnica, mustard, turpentine, etc.

4. *Erythema intertrigo* is a common affection occurring in those parts of the cutaneous surface that are in apposition, as in the axilla, about the thighs, between the nates and under the mammary gland in the female. On the delicate skin of the infant it may often be seen as a result of irritation from the urine and discharges from the bowels, especially if proper cleanliness is not observed. In adults the irritation proceeds mostly from excessive perspiration and is generally encountered in corpulent persons. It may also be caused by purulent discharges from the genital organs in both sexes. When the irritation is kept up for sometime the skin becomes highly congested; the epidermis is macerated, presenting an abraded surface, and in some cases the process goes on to inflammation, giving rise to dermatitis or eczema.

The treatment of this variety should consist in strict attention to cleanliness and in the employment of soothing absorbent agents. The parts in apposition should be kept separated by means of absorbent cotton or lint, and the affected surface kept as dry as possible by the frequent use of dusting-powders, as talc, rice powder and corn starch. Occasional applications of some soothing and slightly astringent lotion are at times beneficial, as oxide of zinc and calamine, each one drachm to rose water four ounces. Symptomatic active hyperæmiæ embrace those erythemas that are the result of constitutional disturbances due to fevers, as typhoid, variola, vaccinia, or to the ingestion of certain drugs, such as belladonna, copaiba, quinine. Also those resulting reflexly, from anger, shame, embarrassment (erythema pudoris). Reflex erythemas may be due to disorders of internal organs, and are common in infancy during the period of dentition. The eruption appears in the form of a diffuse redness or in spots called *roseola* (rose rash.)

Passive hyperæmia is the result of obstruction to the circulation, either peripheral or central, causing a stagnation of the current. The seat of the disturbance is marked by livid, dark red or violaceous discoloration. There are two forms, idiopathic and symptomatic.

Idiopathic passive hyperæmiæ are those conditions produced by direct and long-continued mechanical interference with the circulation, as pressure on the veins from bandages, garters, tightly fitting clothing, etc. This is most commonly met with on the lower extremities. The veins become dilated, and exhibit in extreme cases a tortuous and knotty appearance. Long exposure to a cold atmosphere is also a cause, affecting the fingers, toes, end of the nose and ears. When the general surface of the body is exposed, it produces a mottled appearance. Those lesions that result from the gravitation (hypostases) of blood to dependent parts of the body when long in a recumbent position are also embraced under this head.

Symptomatic passive hyperæmiæ consist of a more or less general bluish discoloration of the skin, called *cyanosis*, and may be due to non-closure of the foramen ovale, to valvular disease, or obstruction on the right side of the heart, or to certain respiratory disorders, as emphysema, pneumonia, pleurisy, tuberculosis, or the presence of tumors that may interfere with the circulation and cause venous stasis. A cyanotic condition often develops as a symptom of asphyxia, due to membranous croup, œdema of the glottis, or any state of suffocation from mechanical obstruction of the air passages. The treatment of most cases of passive hyperæmia is, at most, only palliative. When possible, the cause should be removed. In the lower extremities the roller bandage often gives relief. In cyanosis due to morbid conditions of the cardiac and respiratory apparatus the treatment should be directed to that end. As the dark color is the result of insufficient oxygenation of the blood, it has been thought by some that certain agents given with a view to supplying oxygen to the blood would be beneficial. Thus the peroxide of hydrogen, five to ten drops, has been suggested.

